# Serum biomarkers of liver fibrosis identify changes in striatal metabolite levels

**DOI:** 10.21203/rs.3.rs-2729490/v1

**Published:** 2023-03-28

**Authors:** Natalie Zahr, Edith Sullivan, Adolf Pfefferbaum

**Affiliations:** Stanford University; Stanford University School of Medicine; SRI International Center for Health Sciences

**Keywords:** AST, ALT, platelets, APRI, FIB4, AUD, HIV, CD4 cell count, basal ganglia

## Abstract

^1^H-magnetic resonance spectroscopy (MRS) conducted in cirrhosis shows consistent CNS changes such as high levels of the combined resonances (Glx) of glutamate (Glu) + glutamine (Gln) and low levels of choline-containing compounds (Cho) and myo-Inositol (mI) relative to total creatine (tCr). Studies of hepatitis C virus (HCV) infection, however, note higher than control levels of tCr, Cho, and mI. Here, serum markers of liver fibrosis were evaluated to determine whether they would discriminate neurometabolites in striatum, cerebellum, and pons. An aspartate aminotransferase to platelet ratio index (APRI)>0.7 identified liver fibrosis in 9.0% (n=13) of the cohort; a fibrosis score (FIB4)>1.5 identified liver fibrosis in 32.4% (n=34) of the population. Those with APRI>0.7 had higher levels of striatal tCr (p=.001) and Cho (p=.0003). Similarly, those with FIB>1.5 had higher levels of striatal Cho (p=.01). A multiple regression including the variables APRI>0.7 and HCV explained 16.5% of the variance in striatal Cho and was driven by the APRI. Likewise, the FIB4 relative to HCV explained more of the variance in striatal Cho. Higher striatal Cho levels showed a positive relationship with pallidal signal intensities (r=.18, p=.04). Further, higher pallidal T1-signals were associated with greater standing balance instability with eyes closed (r=−.22, p=.008). Together, these results suggest that elevations in striatal Cho and basal ganglia T1-signal intensities are related to presence of liver fibrosis with functional consequences.

## Introduction

Hepatic encephalopathy (HE), the most well recognized neurological complication of serious liver failure, can manifest as drowsiness, depressive mood, tremor, motor disturbances, cognitive dysfunction, and in later stages, confusion, and coma (Butterworth 2003; Butterworth et al. 2009; Poordad 2007; Losowsky and Scott 1973; Prakash and Mullen 2010; Diaz-Gomez et al. 2011; Ridola et al. 2020; Amodio and Montagnese 2021; Ryu, Rahimi, and Leise 2020; Han et al. 2020; Cheon and Song 2021; Vaquero et al. 2003). In autopsy studies, patients with massive hepatic necrosis were noted to have cerebral edema described as flattening of gyri, partial obliteration of the sulci, compression of lateral ventricles, poor gray and white matter tissue differentiation; and swollen glial cells prominent in both gray and white matter (Ware, D’Agostino, and Combes 1971; Watanabe, Shiota, and Tsuji 1992). Additional support for the presence of cerebral edema came from descriptions of intracranial hypertension in patients who had died of hepatitis (Hanid et al. 1980; Lidofsky et al. 1992). Computed tomographic (CT) (Wijdicks et al. 1995; Donovan et al. 1998) and later magnetic resonance imaging (MRI) studies (Patel et al. 2004; Arnold et al. 2001) confirmed the presence of cerebral edema, particularly in late HE. In vivo diffusion weighted imaging likewise demonstrated changes in metrics [i.e., increases in apparent diffusion coefficients (ADC)] consistent with edema (i.e., swelling due to fluid accumulation) (Poveda et al. 2010; Lodi et al. 2004; Kale et al. 2006; Kumar et al. 2008).

Despite many hypotheses proposed to explain increases in brain fluid in hepatic disease, one that gained the greatest toehold involves the accumulation of glutamine. Indeed, the consistent finding of higher than control levels of the combined resonance of glutamine and glutamate (Glx) relative to total creatine (tCr) – quantified using MR spectroscopy (MRS) in voxels placed in cortical regions of the HE brain – has been used to support the presence of cerebral edema (Binesh et al. 2006; Chavarria et al. 2013; Gupta et al. 1993; Jain et al. 2013; Häussinger et al. 1994; Kreis et al. 1992; Pujol et al. 1996; Ross et al. 1994; Singhal et al. 2010; Thomas et al. 1998). Similar neurometabolic disturbances in cirrhosis absent HE have been interpreted as reflecting “low grade” edema (Geissler et al. 1997; Lee et al. 1999; Ahluwalia et al. ; Mardini et al. 2011; Balata et al. 2003; Cordoba et al. 2001; Laubenberger et al. 1997; Ciecko-Michalska et al. 2012; Meng et al. 2015). The generally accepted mechanism of HE pathology involving high circulating levels of ammonia is supported by the efficacy of lactulose treatment, which decreases the intestinal production and absorption of ammonia (Dhiman et al. 2000; Prasad et al. 2007). Links between high circulating ammonia, cerebral glutamine, and edema are conceptualized as follows. A byproduct of ingested protein metabolism, nitrogen not converted by the liver into urea gets released into general circulation as ammonia (Wijdicks 2016; Butterworth 2014, 2015; Khan and Anjum 2021). In brain, excess ammonia is incorporated into nontoxic glutamine via glutamine synthetase in astrocytes (Yamamoto et al. 1987; Zhou et al. 2020). High intracellular glutamine increases osmotic pressure resulting in swollen astrocytes (Klatzo 1967; Ryan and Shawcross 2015; Kundra et al. 2005; Rama Rao and Norenberg 2014; Takahashi et al. 1991), the postmortem hallmark of “cytotoxic” brain edema (Kundra et al. 2005; Rama Rao and Norenberg 2014; Yamamoto et al. 1987; Liang et al. 2007). The frequently reported decreases in cirrhosis in levels of choline-containing compounds (Cho) and myo-Inositol (mI) relative to tCr are presumed to reflect efforts to main intracellular osmotic gradients (Binesh et al. 2006; Chavarria et al. 2013; Gupta et al. 1993; Jain et al. 2013; Häussinger et al. 1994; Kreis et al. 1992; Pujol et al. 1996; Ross et al. 1994; Singhal et al. 2010; Thomas et al. 1998).

Chronic liver disease is also associated with bilateral, symmetric, high-intensity signals in basal ganglia structures on T1-weighted MRI (Cordoba et al. 2002; Naegele et al. 2000; Binesh et al. 2006; Pujol et al. 1996; Taylor-Robinson et al. 1995). Initially considered a component of HE, T1-signal alterations are now understood as an independent phenomenon reflecting chronic disease rather than acute HE (Weissenborn et al. 1995; Weissenborn et al. 2004). Converging lines of evidence have promoted a mechanism involving manganese (Mn) deposition as contributing to the altered basal ganglia MR signal (Spahr et al. 1996; Krieger et al. 1995; Forton et al. 2004; Rose et al. 1999). It is stipulated that Mn deposition occurs preferentially in basal ganglia structures because they have high levels of glutamine synthetase (Bernstein et al. 2014), which requires Mn (Wedler, Denman, and Roby 1982; Wedler and Denman 1984); as such, Mn deposition reflects an adaptive process to improve ammonia detoxification (i.e., via increased glutamine synthetase activity) (Morgan 1998; Kulisevsky et al. 1992). Despite several multimodal neuroimaging studies describing both T1-weighted and MRS changes in cirrhosis (e.g., Awada et al. 1995; Taylor-Robinson et al. 1995; Krieger et al. 1996; Chavarria et al. 2013; Ahluwalia et al. 2015; Jain et al. 2013; Kreis et al. 1992; Laubenberger et al. 1997), few explored relations between these two neuroimaging markers. In one, a high intensity T1 signal in pallidum was associated with higher Glx/tCr levels (Geissler et al. 1997), but in others, there were no relations between higher signal intensities and changes in MRS metabolites (Catafau et al. 2000; Taylor-Robinson et al. 1996).

Although patients infected with hepatitis C virus (HCV) do not typically present with overt neurological symptoms, complaints such as chronic fatigue and reduced quality of life (Ware et al. 1999; Barkhuizen et al. 1999) prompted in vivo cerebral examination. In HCV, changes in neurometabolite levels contrast with findings in cirrhosis and demonstrate higher than control levels of Cho/tCr and mI/tCr in basal ganglia and white matter regions (Thames et al. 2015; Forton et al. 2001; Forton et al. 2002; McAndrews et al. 2005; Forton et al. 2008; Bokemeyer et al. 2010; Grover et al. 2012; Bladowska et al. 2013). Indeed, recent reviews conclude that in addition to high Cho and mI, tCr levels are also higher in those with than those without HCV infection (Oriolo et al. 2018; Carvalho et al. 2022).

Among clinical diagnoses that can increase the risk for liver disease are alcohol use disorders (AUD) and infection with the human immunodeficiency virus (HIV). When considered as a primary cause and cofactor, alcohol accounts for upwards of 30% of cirrhosis-related deaths globally (Moon, Singal, and Tapper 2020; Stein et al. 2016). Similarly, liver disease remains a leading cause of death among people living with HIV (Sherman, Peters, and Thomas 2017). Further, HCV co-morbidity is prevalent in those with AUD (Singal and Anand 2007; Shoreibah, Anand, and Singal 2014) and HIV (Weber et al. 2006; Pokorska-Śpiewak et al. 2017; Valcour et al. 2016). Here, among a participant sample including those with HIV, AUD, HCV, and their comorbidities, the presence of liver fibrosis [determined using noninvasive serum biomarkers of aspartate aminotransferase to platelet ratio index (APRI) and fibrosis score (FIB4)] was used to distinguish the levels of cerebral metabolites in striatum, cerebellum, and pons. It was hypothesized that the presence of liver fibrosis would predict higher levels of Cho, mI, and tCr in striatum but not the other regions. Further, it was predicted that higher striatal Cho would correlate with higher T1-weighted signals in basal ganglia structures.

## Methods

### Study Participants

This report used data from an existing cohort of study participants examined between March 2007 and July 2010; portions of the data have been published elsewhere (Zahr et al. 2014; Zahr et al. 2013). Data collection was conducted in accordance with protocols approved by the Institutional Review Boards of Stanford University and SRI International including written informed consent from all participants. A total of 144 individuals included healthy controls (n = 38, 47.8±16.7 years), those diagnosed with AUD (n = 36, 48.3±10.4 years; currently sober as demonstrated by a negative Breathalyzer test given immediately after consent), HIV infection (n = 32, 50.8±8.1 years), and AUD + HIV (n = 38, 50.3±7.7 years).

Participants were screened using the Structured Clinical Interview for the Diagnostic Statistical Manual [(DSM-IV) SCID] (First et al. 1998), structured health questionnaires, and a semi-structured timeline follow-back interview to quantify lifetime alcohol consumption (Skinner and Sheu 1982). Upon initial assessment, subjects were excluded if they had a significant history of medical (e.g., epilepsy, stroke, multiple sclerosis, uncontrolled diabetes, or loss of consciousness > 30 minutes), psychiatric (e.g., schizophrenia, bipolar disorder), or neurological (e.g., Parkinson’s disease) disorders other than DSM-IV alcohol abuse or dependence (herein designated AUD). Other exclusionary criteria were substance dependence (other than alcohol for the 2 AUD groups) within the past 3 months or any other DSM disorder (for all 4 groups). The SCID interview also provided a Global Assessment of Functioning (GAF) score, which is a single rating scale of overall functioning ranging from 1 for sickest to 100 for healthiest individuals (Endicott et al. 1976; Williams and Rabkin 1991). Socioeconomic status (SES) was derived from the *Four-Factor Index of Social Status*, which considers education and occupation level and wherein a lower score reflects higher status (Hollingshead 1975).

The 4 groups were well-matched with respect to age, handedness, and body mass index (BMI, **Table 1**). As in other studies, the 3 diagnostic groups relative to the healthy control group had fewer years of education, lower SES, and lower GAF scores than controls (all p < .0001) (Zahr et al. 2021; Sullivan et al. 2018; Pfefferbaum et al. 2018). Further, the 3 diagnostic groups relative to the healthy control group were more likely to include African American or Black individuals (p = .0002), people who smoke (p < .0001), and be seropositive for hepatitis C virus (HCV, p < .0001).

### Blood Sample Collection and Liver Fibrosis Biomarkers

Serum samples were collected and analyzed by Quest Diagnostics for complete blood count (CBC) with differential, comprehensive metabolic panel, and HIV and HCV screening with RNA quantification for seropositive individuals. Laboratory results were used to calculate 2 validated, non-invasive indices of liver injury: the APRI, based on aspartate aminotransferase (AST) and platelet levels (Thandassery et al. 2016; Fouad et al. 2012) and the FIB4, based on age, AST, alanine aminransferase (ALT), and platelet levels (Sterling et al. 2006).


$$\text{ APRI }=\frac{\left(\frac{\text{AST}\left(\frac{IU}{L}\right)}{\text{ AST (upper limit of normal) }\left(\frac{IU}{L}\right)}\right)}{\text{ platelet count }\left(10^{9}/L\right)}*100$$

$$\text{FIB}4=\frac{\text{age}(\text{ years })*\text{AST}\left(\frac{IU}{L}\right)}{\text{ platelet count }\left(10^{9}/L\right)*\sqrt{\text{ALT}\left(\frac{IU}{L}\right)}}$$

An APRI > 1.5 predicts cirrhosis; a score > 0.7 has a sensitivity of 77% and specificity of 72% for predicting significant hepatic fibrosis (Lin et al. 2011; Chou and Wasson 2013; McPherson et al. 2010). A FIB4 > 3.25 predicts advanced cirrhosis, while scores > 1.5 indicate fibrosis (McPherson et al. 2010; Sterling et al. 2006; Lavender et al. 2020).

### MR Imaging (MRI) Acquisition and Analysis

MR data were collected and processed using an in-house pipeline (Pfefferbaum et al. 2018). Briefly, data were collected on a 3-Tesla GE whole-body MR system (General Electric Healthcare, Waukesha, WI) using an 8-channel phased-array head coil. The T1-weighted sequence was an axial Inversion-Recovery Prepared SPoiled Gradient Recalled (SPGR) (repetition time (TR) = 6.55, echo time (TE) = 1.56, inversion time (TI) = 300 ms, matrix = 256×256, thickness = 1.25 mm, skip = 0 mm, 124 slices, field of view (FOV) = 24cm). An axial fast spin echo (FSE) T2-weighted MRI (field of view = 24 cm, frequency encode = 256, average echo time (TE)1/TE2/ repetition time (TR) = 17/102/7500 ms, phase encode = 192, echo train length = 8, slice thickness = 2.5 mm, spacing = 0mm) was used for MRS voxel placement.

Preprocessing of T1-weighted SPGR data involved noise removal (Coupe et al. 2008) and brain mask segmentation using FSL BET (Smith 2002), AFNI 3dSkullStrip (Cox 1996), and Robust Brain Extraction (ROBEX) (Iglesias et al. 2011) generating 3 brain masks. In parallel, noise-corrected, T1-weighted images were corrected for field inhomogeneity via N4ITK (Avants et al. 2011), brain masks were segmented (Sadananthan et al. 2010), and the resulting segmented brain masks were reduced to one using majority voting (Rohlfing et al. 2004). Brain tissue segmentation (gray matter, white matter, and cerebrospinal fluid [CSF]) of the skull-stripped T1-weighted images was generated via Atropos (Avants et al. 2011). Parcellated maps of tissue used the parc116 atlas to define cortical (gray matter) and subcortical (gray and white matter) regions of interest (ROI). For signal intensity calculations, ROI segmentations were overlaid on T1-weighted images. T1-weighted signal intensities per ROI as ratios relative to pontine T1-weighted signal intensity were calculated for 3 basal ganglia structures (caudate, putamen, globus pallidus) and thalamus.

### MR Spectroscopy (MRS) Acquisition and Analysis

**Figure 1:**
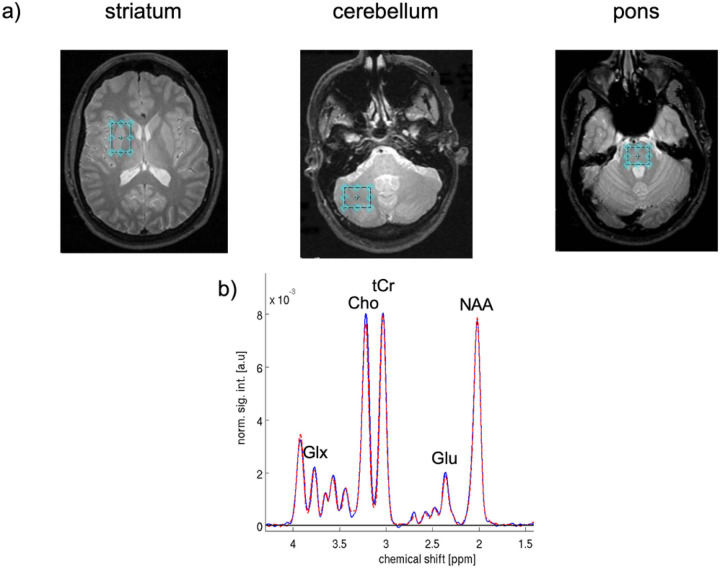
Exemplary voxel placement in striatum, cerebellum, and pons (top) and spectra averaged from the control subjects in the striatal voxel (bottom).

MRS was performed using constant time point-resolved spectroscopy (CT-PRESS)(Dreher and Leibfritz 1999). Single voxels were manually positioned in left or right striatum (10.6 cc), left or right cerebellum(9.8 cc), and central pons (5.9 cc); hemisphere of voxel placement was balanced across subjects and groups (Fig. 1a). The acquisition time was ~ 9 min per voxel (TE = 139ms, 129 chemical shift (CS) encoding steps, *Δt*_*1*_ */2* = 0.8 ms, TR = 2 s, 2 averages)(Mayer and Spielman 2005). A scan without water suppression was acquired (17 CS encoding steps, *Δt*_*1*_ */2* = 6.4 ms, 2 averages) to measure the tissue water content used to normalize the metabolite signal intensities. Data acquired without water suppression were apodized in t_2_ with a 5 Hz Gaussian line broadening and zero-filled up to 4K points for each TE.

After performing a fast Fourier transform (FFT) along t_2_, water spectra were evaluated by peak integration. The amount of CSF and tissue water was estimated by fitting the data across the 17 TEs to a bi-exponential model (Mayer et al. 2007). Apodization of the water-suppressed data entailed multiplication with sine-bell functions in both time dimensions and zero-filling up to 4Kx1K data points. After performing a 2D FFT, effectively decoupled 1D CT-PRESS spectra were obtained by integrating the 2D spectrum in magnitude mode along f_2_ within a ± 13 Hz interval around the spectral diagonal. The quality of the spectra allowed evaluation of signals of the major proton metabolites: N-acetyl aspartate (NAA 2.01 ppm), tCr (3.03 ppm and 3.93 ppm), Cho (3.20 ppm), Glu (2.35 ppm), and mI (3.52 ppm) (Fig. 1b). After baseline subtraction, three large singlet resonances (NAA, tCr, and Cho) were fit simultaneously, and the Glu and mI resonances fit independently, with a Gaussian function within a ± 7.95Hz window using a downhill simplex method (IDL AMOEBA). The integrated area under the fitted Gaussian was used for quantification.

Accuracy of voxel placement is potentially affected by subject motion in the time interval between voxel placement and acquisition as well as during acquisition itself, which can result in reduced spatial overlap between the prescribed and the actual voxel volume. To exclude voxels with significantly reduced overlap, subject motion was detected by comparison of the FSE images acquired before MRS acquisition and a three-plane anatomical localizer acquired thereafter. To this end, binary masks of the prescribed voxel volumes were first generated in the space of the FSE images. The FSE images were then aligned with the post-MRS localizer by rigid image registration (http://nitrc.org/projects/cmtk). If there was no motion, the resulting image-to-image coordinate transformation should be identical to the ideal transformation, which is determined from the image coordinates in the scanner coordinate system as recorded in the DICOM image files. The deviation of the actual from the ideal transformation (i.e., the difference transformation) represents the subject motion between the FSE used for voxel prescription and the post-MRS localizer scan. The effect of motion on a voxel depends not only on the detected motion but also on the location and size of that voxel (e.g., rotation around the center of a voxel has a smaller effect than rotation by the same angle around a point outside the voxel). To quantify this effect, the difference transformation was applied to each prescribed voxel ROI in FSE space, yielding a reformatted voxel ROI, also in FSE space, which represented the voxel that was acquired. The overlap of prescribed and acquired voxel was then computed and expressed in percent of the voxel volume. When there was no motion detected, the difference transformation was the identity mapping and both ROIs were identical, resulting in perfect 100% overlap. A threshold for partitioning of the overlap distribution at 62% for all three regions was used.

A total of 7 striatal (5 poor spectral quality, 2 low overlap; 3 AUD, 3 HIV, 1 AUD + HIV), 10 cerebellar (7 poor spectral quality, 3 low overlap; 3 control, 5 AUD, 2 AUD + HIV), and 17 pontine (9 poor spectral quality, 6 low overlap, 2 never collected; 4 control, 8 AUD, 2 HIV, 3 AUD + HIV) voxels were excluded.

### Upper Motor, Ataxia, and Fluency Testing

Scores were available for upper motor tests of fine finger movement (sum of all conditions) (Fama et al. 2007) and grooved pegboard (sum of left- and right-hand) (Trites 1977). Postural balance was evaluated using the walkaline ataxia battery, conducted first with eyes open and then with eyes closed under 4 conditions: stand heel-to-toe on a line with arms folded across the chest for a maximum of 60s; walk a line heel to-toe for 10 steps; stand on the left foot for 30s; stand on the right foot for 30s. Each was conducted twice unless the subject achieved a perfect score on the first trial, in which case the second trial was also given a perfect score. Scores were summed for the eyes open or closed conditions (Fregly, Graybiel, and Smith 1972; Sullivan et al. 2000). Verbal facility was assessed using performance on phonological fluency (letters F, A, and S) (Spreen 1977) and semantic fluency (inanimate objects, animals, birds/colors) (Martin et al. 1990).

## Statistical Analysis

Statistics were performed using JMP^®^ Pro 16.0.0 (SAS Institute Inc., Cary, NC, 1989–2021). Analysis of variance (ANOVA) was used for 4 group comparisons (Table 1); two-group comparisons used χ^2^ or t-tests as appropriate (Tables 1 & 3). Welch’s test was used for comparison of groups with unequal variances (Tables 3 & 4). Multiple regression models were used to account for variance from correlated variables (Table 5). Correlations were evaluated using simple linear regressions (Table 6) or nonparametric Spearman’s ρ (Table 7). Significance required Bonferroni-corrected p-values: for demographics, p≤.006 (i.e., .05/8, the number of basic demographic variables in Tables 1 & 3); for metabolites, p≤.01 (i.e., .05/5 metabolites per voxel, Table 4); for T1-weighted signal intensities p≤.01 (i.e., .05/4 regions), for performance, p≤.008 (i.e., .05/6, the number of tests evaluated, Table 7).

## Results

### Serum Fibrosis Scores

Using the APRI cutoff, 9.0% (n = 13) of the sample had serum signs of fibrosis (1 control, 2 individuals with AUD, 4 with HIV, and 6 with AUD + HIV); the FIB4 > 1.5 cutoff identified 23.6% (n = 34) of the cohort as having serum signs of fibrosis (5 controls, 5 individuals with AUD, 13 with HIV, and 11 with AUD + HIV, **Table 2**). Demographic characteristics by fibrosis cutoff scores or presence of HCV are presented in **Table 3**. Of note, collapsed across the original diagnoses, individuals with APRI > 0.7 were more likely than those below the cutoff to have a lower current CD4 cell count (F = 11.0, p = .0038) and to be seropositive for HCV (χ^2^ = 14.9, p = .0001); those with FIB > 1.5 were older (F = 18.8, p < .0001) and more likely to be seropositive for HCV (χ^2^ = 18.5, p < .0001).

### CNS Metabolite Levels

**Figure 2:**
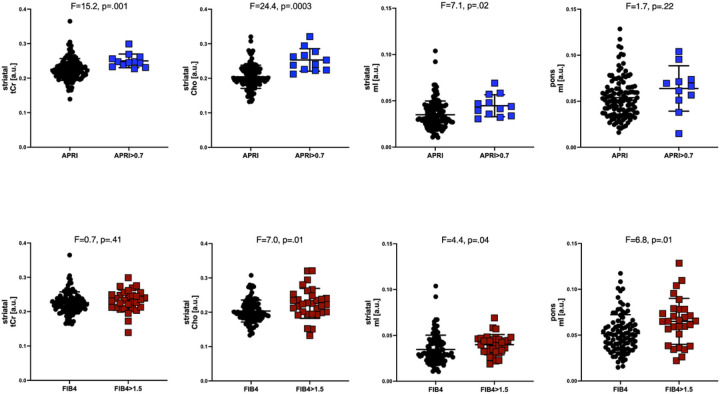
Differences in levels of striatal tCr, Cho, mI, and pontine mI as a function of APRI>0.7 (top) or FIB>1.5 (bottom).

The 5 metabolites in each of 3 brain regions were evaluated by fibrosis cutoff scores and HCV status (**Table 4**). In those with APRI scores above relative to those with scores below the cutoff, levels of striatal tCr (F = 15.1, p = .0012) and Cho (F = 24.4, p = .0003, Fig. 2) were higher. Similarly, in those with FIB4 fibrosis relative to those without fibrosis, levels of striatal Cho (F = 7.0, p = .01) were high as were levels of pontine mI (F = 6.8, p = .01, Fig. 2). Those seropositive for HCV relative to those without HCV infection had higher striatal Cho (F = 7.1, p = .01) and Glu (F = 8.4, p = .005) and higher pontine mI (F = 14.8, p = .0003).

The effects of demographic variables on metabolites levels were also considered. Four-group ANOVAs approached a p = .05 but not Bonferroni-corrected significance for mI in striatum (F_3,137_=2.4, p = .07) and pons (F_3,127_=2.4, p = .07), suggesting that the original diagnoses (i.e., AUD, HIV, AUD + HIV) do not distinguish metabolite levels. Metabolites showed both higher and lower levels with older age: across the entire sample, older age was associated with lower levels of striatal NAA (r=−.33, p < .0001) and tCr (r=−24, p = .004); and higher levels of pontine Cho (r = .31, p = .0004) and mI (r = .24, p = .006). Striatal Glu (t=−3.2, p = .002), cerebellar NAA (t=−2.9, p = .005) and Glu (t=−3.4, p = .0009), and pontine NAA (t=−3.3, p = .002) were higher in women than men irrespective of diagnostic group.

For each metabolite showing relations with fibrosis scores (striatal tCr and Cho, pontine mI), multiple regression analyses were used to determine variables significantly related to metabolite changes. That is, each metabolite (e.g., striatal Cho) was evaluated using 2 different multiple regressions: the first model included 5 variables (nominal variables “diagnosis”, “APRI > 0.7”, and “HCV” and the continuous variables “CD4 count” and “age”); the second model included 4 variables (nominal variables “diagnosis”, “FIB > 1.5”, and “HCV” and the continuous variable “age”). The 5 variable model explained 17.2% of the variance in striatal Cho (F_4,99_=3.2, p = .007) driven by APRI > 0.7 (p = .002, **Table 5**). The results were similar when using the FIB4: the 4 variables (diagnosis, FIB4 > 1.5, HCV, age) together explained 11.7% of the variance in striatal Cho in a significant model (F_3,137_=2.9, p = .01) driven by FIB4 > 1.5 (p = .01, **Table 5**). Multiple regression results using APRI model were not significant for striatal tCr; using the FIB4, the model for striatal tCr was significant (F_3,137_=2.6, p = .02) and driven by age (p = .0006). Both models suggested that pontine mI levels were driven by the presence of HCV [i.e., the 5 variable model explained 20.5% of variance in pontine mI (F_4,93_=3.7, p = .003) driven by HCV (p = .01) and age (p = .01); the 4 variable model explained 15.4% of the variance in pontine mI in a model (F_3,127_=3.6, p = .002) driven by HCV (p = .04)].

### MRS Metabolite and T1-weighted Signal Intensity Relations

**Figure 3:**
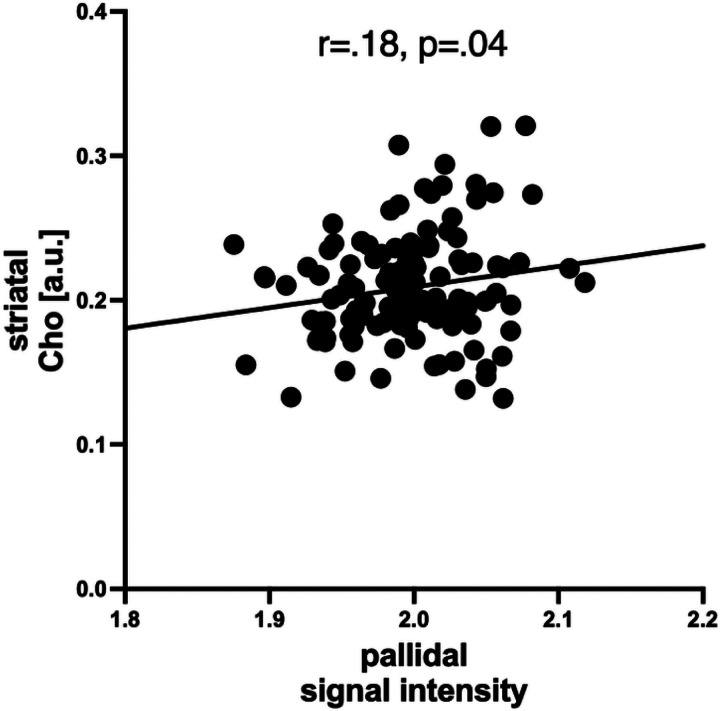
Correlation between higher pallidal signal intensity and greater striatal Cho.

Those with an APRI > 0.7 relative to those with scores below cutoffs had higher T1-weighted signal intensities in caudate (F = 9.2, p = .007) and putamen (F = 10.6, p = .006) and approached significantly higher T1-weighted signal intensities in pallidum (F = 4.1, p = .06) but not thalamus (F = 2.7, p = .11). Similarly, FIB4 > 1.5 distinguished higher T1-weighted signal intensities in caudate (F = 10.6, p = .002), putamen (F = 6.5, p = .01), and pallidum (F = 9.2, p = .004) but not thalamus (F = 1.9, p = .17). The 3 metabolites that showed significant differences in levels due to the presence of liver fibrosis (i.e., striatal tCr and Cho, pontine mI) were evaluated for their relations to signal intensities in caudate, putamen, pallidum, and thalamus. The only significant relation was between higher signal intensity in the pallidum and higher striatal Cho (r = .18, p = .04, Fig. 3).

### CNS Marker and Behavioral Performance Relations

**Figure 4:**
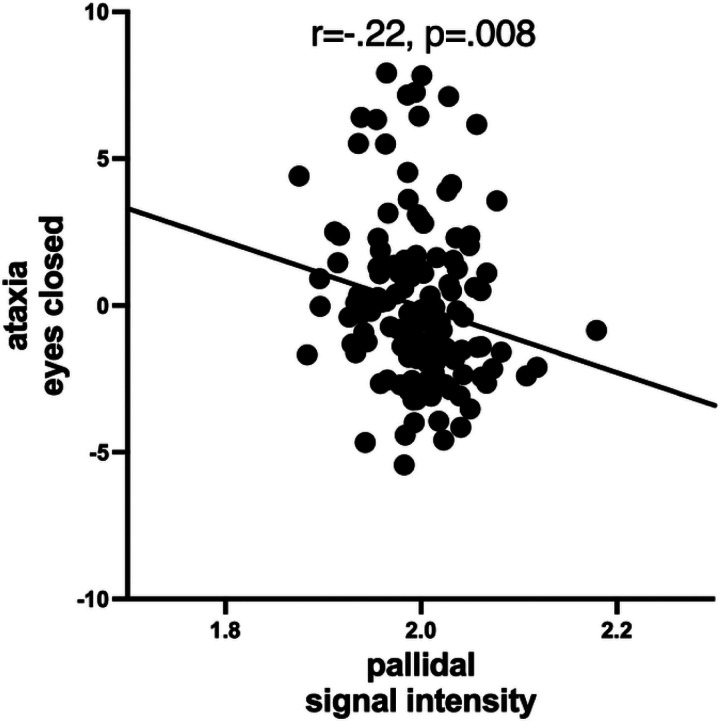
Correlation between higher pallidal signal intensity and worse ataxia performance (eyes closed).

Striatal Cho levels were not associated with performance on any of the motor or fluency tests (**Table 7**). By contrast, higher pallidal signal intensities were associated with worse balance on the ataxia test performed with eyes closed (ρ=−.22, p = .008) (Fig. 4).

## Discussion

We recently demonstrated that fibrosis determined by APRI or FIB4 cutoff scores is associated with CNS changes including higher T1-weighted signal intensities in basal ganglia structures associated with smaller volume selective to the globus pallidus (Kwong and Zahr 2023). Here we demonstrate that in people with APRI or FIB4 identified fibrosis relative to those without serum biomarkers of fibrosis, levels of striatal Cho and tCr and pontine mI are high. These findings are consistent with the literature demonstrating higher than control levels of Cho, tCr, and mI in various ROIs, including basal ganglia, in individuals with HCV (Forton et al. 2001; Forton et al. 2002; McAndrews et al. 2005; Reichardt et al. 2022). The current study, however, extends the literature by suggesting that liver fibrosis and not HCV per se is responsible for the elevation in striatal Cho, whereas elevations in mI and tCr may be better explained by the presence of HCV (cf., Table 5). This interpretation comports with a study that used APRI cutoffs to determine neuropsychological performance in those with HIV or HCV and concluded that liver fibrosis has a contribution to cognitive performance independent of HCV and HIV (Valcour et al. 2016). Here we suggest that liver fibrosis is associated with high levels of striatal Cho independent of HCV, HIV, or AUD. Longitudinal studies including people with liver disease from multiple etiologies and at earlier disease stages will be necessary to determine whether an increase in Cho is a feature of early, pre-cirrhotic liver disease whereas reduced Cho occurs in later liver disease stages (cf., Geissler et al. 1997; Lee et al. 1999; Ahluwalia et al. ; Mardini et al. 2011; Balata et al. 2003; Cordoba et al. 2001; Laubenberger et al. 1997; Ciecko-Michalska et al. 2012; Meng et al. 2015).

The current study also demonstrated that higher levels of striatal Cho are associated with a higher pallidal T1 signal. The hyperintense pallidal signal observed in cirrhosis is unusual in that it is present on T1- but not T2- weighted images and is not vulnerable to gadolinium enhancement (Pujol et al. 1993; Awada et al. 1995; Zeneroli et al. 1991; Brunberg et al. 1991). This signal was initially proposed to arise from the deposition of paramagnetic substances such as Mn (Inoue et al. 1991; Newland et al. 1989) or to lipid accumulation (Young 1984) because these are among the few naturally occurring substances that are known to reduce T1 relaxation times (Ginat and Meyers 2012) without affecting T2-weighted imaging (i.e., calcification or hemorrhaging would affect both T1 and T2 signaling). Due to converging lines of evidence, the mechanism involving Mn deposition as contributing to the altered pallidal T1-signal gained the greatest traction (Spahr et al. 1996; Krieger et al. 1995; Forton et al. 2004; Rose et al. 1999). Caveats regarding Mn deposition as the underlying mechanism of pallidal T1-weighted signal changes include the following considerations: systemic Mn levels are not related to pallidal signal intensity (Maffeo et al. 2014; Krieger et al. 1996); in iron-deficiency anemia, serum Mn levels are high but the pallidal signal is not present (Kim et al. 2005); and postmortem examination of tissue from patients with liver disease reveals Mn deposition beyond the pallidum in caudate, putamen, substantia nigra, and cerebellum (Klos et al. 2006; Krieger et al. 1995) and high levels of other metals such as copper in similar regions (Maeda et al. 1997). Further, animal exposure studies do not show preferential Mn loading to basal ganglia structures (Takeda, Sawashita, and Okada 1998; Finkelstein et al. 2008); notable Mn accumulation also occurs in olfactory bulb, frontal cortex, hippocampus, and cerebellum (Elder et al. 2006; Finkelstein et al. 2008; Ma et al. 2018; Fitsanakis et al. 2008; Pautler, Mongeau, and Jacobs 2003; Long et al. 2014). Indeed, an unresolved question with respect to the Mn hypothesis is a mechanistic explanation for preferential deposition in the pallidum.

Iron induced, oxidative damage to lipids is a proposed mechanism of liver disease pathophysiology (Britton 1996; Britton, Subramaniam, and Crawford 2016; Ahmed, Latham, and Oates 2012). A similar mechanism may explain pallidal susceptibility to chronic liver disease. The globus pallidus is unique among brain regions in having high iron and high myelin content: in healthy basal ganglia, high iron accounts for a hypointense pallidum on T2-weighted images, whereas the caudate and putamen are isointense relative to cortical gray matter (Hegde et al. 2011); a high pallidal T1-signal relative to other basal ganglia regions is associated with its greater myelin content (Henkelman, Watts, and Kucharczyk 1991; Van Cauter et al. 2020; Zaitout et al. 2014). Iron-induced damage of pallidal myelin may result in the accumulation of lipids (i.e., damaged biological membranes) which can accentuate water proton relaxation and contribute to increasing the pallidal T1-weighted signal {Brunberg, 1991 #9741;Warakaulle, 2003 #9792;Lai, 1999 #9786;Young, 1984 #9638;Gupta, 2017 #9789; Kucharczyk, 1990 #9874;Ginat, 2012 #11087}. As the Cho signal arises from choline-containing compounds such as membrane phospholipids and as Cho elevations are consistent with cell membrane turnover (Adalsteinsson, Sullivan, and Pfefferbaum 2002; Mader et al. 2008), a significant positive relationship between striatal Cho and the pallidal T1-weighted signal may signify impaired membrane homeostasis that may precede demyelination in chronic liver disease {Hathout, 2015 #11127}. Indeed, moderate myelin loss and lipidic droplets were observed in postmortem pallidal tissue in those who exhibited altered pallidal signals in vivo {Kulisevsky, 1992 #9731}. Further support for the hypothesis that disruption of myelin homeostasis represented by high levels of Cho may underlie pallidal T1 changes in liver disease will require larger, longitudinal neuroimaging studies across the liver disease time course.

High pallidal signal intensities in cirrhosis have been associated with excessive postural body sway (Kim et al. 2007), tremor of the hands (Pujol et al. 1993), and impaired performance on grooved pegboard (Chang et al. 2009; Chang et al. 2010; Kulisevsky et al. 1992), but consistent functional consequences of T1-signal alterations have not been forthcoming (cf., Shin and Park 2017). In our recent study, higher pallidal signal intensity was associated with greater postural instability in both eyes open and closed conditions (Kwong and Zahr 2023). Here, consistent with our own previous finding and the literature, higher pallidal signal intensities were associated with ataxia with eyes closed.

Together, previous MRS results demonstrating changes to tCr in HCV (Bladowska et al. 2013; McAndrews et al. 2005), ^31^phosphoros MRS experiments demonstrating bioenergetic abnormalities (e.g., increase in phosphocreatine) in patients with minimal HE or stable overt chronic HE (Patel et al. 2000), and the current results suggest that tCr should not be used as a referent in MRS studies of liver disease.

Limitations of the current study include the relatively small number of individuals with APRI-defined fibrosis (n = 13) and the inclusion of those with liver fibrosis due to several etiologies (i.e., AUD, HIV, HCV). Indeed, there is some evidence suggesting that CNS changes could be unique to liver disease etiology (e.g., alcohol vs. non-alcohol related cirrhosis) (Miese et al. 2006). Further, although we demonstrate that the effects of fibrosis on striatal Cho are greater than the effects of HCV per se, the relatively high representation of HCV in the APRI and FIB4 groups precludes a clear dissociation. Finally, elevated levels of Cho have been interpreted as reflecting energy failure (Zahr et al. 2014; Brooks et al. 2000; Callot et al. 2008; Bizzi et al. 2001; Fischer-Smith et al. 2004) and may not reflect membrane degradation or demyelination as suggested here.

In conclusion, using serum biomarkers of liver fibrosis, higher than control values of Cho were identified in striatum and related to the pallidal T1-weighted signal providing initial evidence for an alternative mechanism to Mn deposition to explain focal signal brightening in liver disease that may instead arise from accumulation of lipids related to myelin disruption.

## Data Availability

The data that support these findings of this study will be openly available at https://data.mendeley.com/.

## References

[1] Adalsteinsson E., Sullivan E.V., and Pfefferbaum A.. 2002. ‘Biochemical, Functional and Microstructural Magnetic Resonance Imaging (MRI).’ in Liu Y. and Lovinger D. M. (eds.), Methods in Alcohol-Related Neuroscience Research (CRC Press: Boca Raton).

[2] Ahluwalia V., Wade J. B., Moeller F. G., White M. B., Unser A. B., Gavis E. A., Sterling R. K., Stravitz R. T., Sanyal A. J., Siddiqui M. S., Puri P., Luketic V., Heuman D. M., Fuchs M., Matherly S., and Bajaj J. S.. 2015. ‘The etiology of cirrhosis is a strong determinant of brain reserve: A multimodal magnetic resonance imaging study’, Liver Transpl, 21: 1123–32. 25939692 10.1002/lt.24163 PMC4550553

[3] Ahmed U., Latham P. S., and Oates P. S.. 2012. ‘Interactions between hepatic iron and lipid metabolism with possible relevance to steatohepatitis’, World J Gastroenterol, 18: 4651–8. 23002334 10.3748/wjg.v18.i34.4651 PMC3442203

[4] Amodio P., and Montagnese S.. 2021. ‘Lights and Shadows in Hepatic Encephalopathy Diagnosis’, J Clin Med, 10. 10.3390/jcm10020341 PMC7831133 33477554

[5] Arnold S. M., Els T., Spreer J., and Schumacher M.. 2001. ‘Acute hepatic encephalopathy with diffuse cortical lesions’, Neuroradiology, 43: 551–4. 11512584 10.1007/s002340000461

[6] Avants B. B., Tustison N. J., Song G., Cook P. A., Klein A., and Gee J. C.. 2011. ‘A reproducible evaluation of ANTs similarity metric performance in brain image registration’, Neuroimage, 54: 2033–44. 20851191 10.1016/j.neuroimage.2010.09.025 PMC3065962

[7] Awada A., Sullivan S., Palkar V., Sbeih F., Naufal R., and Al Rajeh S.. 1995. ‘Brain magnetic resonance imaging in non-alcoholic cirrhosis’, Eur J Radiol, 21: 84–8. 8850497 10.1016/0720-048x(95)00694-l

[8] Balata S., Olde Damink S. W., Ferguson K., Marshall I., Hayes P. C., Deutz N. E., Williams R., Wardlaw J., and Jalan R.. 2003. ‘Induced hyperammonemia alters neuropsychology, brain MR spectroscopy and magnetization transfer in cirrhosis’, Hepatology, 37: 931–9. 12668989 10.1053/jhep.2003.50156

[9] Barkhuizen A., Rosen H. R., Wolf S., Flora K., Benner K., and Bennett R. M.. 1999. ‘Musculoskeletal pain and fatigue are associated with chronic hepatitis C: a report of 239 hepatology clinic patients’, Am J Gastroenterol, 94: 1355–60. 10235218 10.1111/j.1572-0241.1999.01087.x

[10] Bernstein H. G., Bannier J., Meyer-Lotz G., Steiner J., Keilhoff G., Dobrowolny H., Walter M., and Bogerts B.. 2014. ‘Distribution of immunoreactive glutamine synthetase in the adult human and mouse brain. Qualitative and quantitative observations with special emphasis on extra-astroglial protein localization’, J Chem Neuroanat, 61–62: 33–50. 10.1016/j.jchemneu.2014.07.003 25058171

[11] Binesh N., Huda A., Thomas M. A., Wyckoff N., Bugbee M., Han S., Rasgon N., Davanzo P., Sayre J., Guze B., Martin P., and Fawzy F.. 2006. ‘Hepatic encephalopathy: a neurochemical, neuroanatomical, and neuropsychological study’, J Appl Clin Med Phys, 7: 86–96. 10.1120/jacmp.v7i1.2151 PMC5722484 16518320

[12] Bizzi A., Ulug A. M., Crawford T. O., Passe T., Bugiani M., Bryan R. N., and Barker P. B.. 2001. ‘Quantitative proton MR spectroscopic imaging in acute disseminated encephalomyelitis’, AJNR Am J Neuroradiol, 22: 1125–30. 11415908 PMC7974787

[13] Bladowska J., Zimny A., Knysz B., Malyszczak K., Koltowska A., Szewczyk P., Gasiorowski J., Furdal M., and Sasiadek M. J.. 2013. ‘Evaluation of early cerebral metabolic, perfusion and microstructural changes in HCV-positive patients: a pilot study’, J Hepatol, 59: 651–7. 23680314 10.1016/j.jhep.2013.05.008

[14] Bokemeyer M., Ding X. Q., Goldbecker A., Raab P., Heeren M., Arvanitis D., Tillmann H. L., Lanfermann H., and Weissenborn K.. 2010. ‘Evidence for neuroinflammation and neuroprotection in HCV infection-associated encephalopathy’, Gut, 60: 370–7. 20926642 10.1136/gut.2010.217976

[15] Britton L. J., Subramaniam V. N., and Crawford D. H.. 2016. ‘Iron and non-alcoholic fatty liver disease’, World J Gastroenterol, 22: 8112–22. 27688653 10.3748/wjg.v22.i36.8112 PMC5037080

[16] Britton R. S. 1996. ‘Metal-induced hepatotoxicity’, Semin Liver Dis, 16: 3–12. 8723319 10.1055/s-2007-1007214

[17] Brooks W. M., Stidley C. A., Petropoulos H., Jung R. E., Weers D. C., Friedman S. D., Barlow M. A., Sibbitt W. L. Jr., and Yeo R. A.. 2000. ‘Metabolic and cognitive response to human traumatic brain injury: a quantitative proton magnetic resonance study’, J Neurotrauma, 17: 629–40. 10972240 10.1089/089771500415382

[18] Brunberg James A, Kanal E, Hirsch W, and Van Thiel DH. 1991. ‘Chronic acquired hepatic failure: MR imaging of the brain at 1.5 T’, American journal of neuroradiology, 12: 909–14. 1950920 PMC8333503

[19] Butterworth R. F. 2003. ‘Hepatic encephalopathy--a serious complication of alcoholic liver disease’, Alcohol Res Health, 27: 143–5. 15303624 PMC6668889

[20] ———. 2014. ‘Hepatic encephalopathy in alcoholic cirrhosis’, Handb Clin Neurol, 125: 589–602. 25307598 10.1016/B978-0-444-62619-6.00034-3

[21] ———. 2015. ‘Pathogenesis of hepatic encephalopathy and brain edema in acute liver failure’, J Clin Exp Hepatol, 5: S96–s103. 26041966 10.1016/j.jceh.2014.02.004 PMC4442857

[22] Butterworth R. F., Norenberg M. D., Felipo V., Ferenci P., Albrecht J., Blei A. T., and Ishen Commission on Experimental Models of H. E. Members of the. 2009. ‘Experimental models of hepatic encephalopathy: ISHEN guidelines’, Liver Int, 29: 783–8. 19638106 10.1111/j.1478-3231.2009.02034.x

[23] Callot V., Galanaud D., Le Fur Y., Confort-Gouny S., Ranjeva J. P., and Cozzone P. J.. 2008. ‘(1)H MR spectroscopy of human brain tumours: a practical approach’, Eur J Radiol, 67: 268–74. 18406554 10.1016/j.ejrad.2008.02.036

[24] Carvalho T. L., Mertens Brainer de Queiroz Lima A. C., de Araújo N. S., de Sousa Fernandes M. S., Lira G. B., de Melo M. M. M., Vasconcelos L. R. S., de Moura Pmmf, and da Cunha Correia C.. 2022. ‘Aspects of cognitive assessments and spectroscopic magnetic resonance imaging in people with chronic hepatitis C: a systematic review’, Psychol Health Med: 1–17. 10.1080/13548506.2022.2029915 35075963

[25] Catafau A. M., Kulisevsky J., Bernà L., Pujol J., Martin J. C., Otermin P., Balanzó J., and Carrió I.. 2000. ‘Relationship between cerebral perfusion in frontal-limbic-basal ganglia circuits and neuropsychologic impairment in patients with subclinical hepatic encephalopathy’, J Nucl Med, 41: 405–10. 10716310

[26] Chang Y., Kim Y., Woo S. T., Song H. J., Kim S. H., Lee H., Kwon Y. J., Ahn J. H., Park S. J., Chung I. S., and Jeong K. S.. 2009. ‘High signal intensity on magnetic resonance imaging is a better predictor of neurobehavioral performances than blood manganese in asymptomatic welders’, Neurotoxicology, 30: 555–63. 19376157 10.1016/j.neuro.2009.04.002

[27] Chang Y., Woo S. T., Kim Y., Lee J. J., Song H. J., Lee H. J., Kim S. H., Lee H., Kwon Y. J., Ahn J. H., Park S. J., Chung I. S., and Jeong K. S.. 2010. ‘Pallidal index measured with three-dimensional T1-weighted gradient echo sequence is a good predictor of manganese exposure in welders’, J Magn Reson Imaging, 31: 1020–6. 20373449 10.1002/jmri.22104

[28] Chavarria L., Alonso J., Garcia-Martinez R., Simon-Talero M., Ventura-Cots M., Ramirez C., Torrens M., Vargas V., Rovira A., and Cordoba J.. 2013. ‘Brain magnetic resonance spectroscopy in episodic hepatic encephalopathy’, J Cereb Blood Flow Metab, 33: 272–7. 23168529 10.1038/jcbfm.2012.173 PMC3564202

[29] Cheon S. Y., and Song J.. 2021. ‘The Association between Hepatic Encephalopathy and Diabetic Encephalopathy: The Brain-Liver Axis’, Int J Mol Sci, 22. 10.3390/ijms22010463 PMC7796499 33466498

[30] Chou R., and Wasson N.. 2013. ‘Blood tests to diagnose fibrosis or cirrhosis in patients with chronic hepatitis C virus infection: a systematic review’, Ann Intern Med, 158: 807–20. 23732714 10.7326/0003-4819-158-11-201306040-00005

[31] Ciecko-Michalska I., Dziedzic T., Banys R., Senderecka M., Binder M., Wyczesany M., Szewczyk J., Wojcik J., Slowik A., and Mach T.. 2012. ‘Does magnetic resonance spectroscopy identify patients with minimal hepatic encephalopathy?’, Neurol Neurochir Pol, 46: 436–42. 23161187 10.5114/ninp.2012.31353

[32] Cordoba J., Alonso J., Rovira A., Jacas C., Sanpedro F., Castells L., Vargas V., Margarit C., Kulisewsky J., Esteban R., and Guardia J.. 2001. ‘The development of low-grade cerebral edema in cirrhosis is supported by the evolution of (1)H-magnetic resonance abnormalities after liver transplantation’, J Hepatol, 35: 598–604. 11690705 10.1016/s0168-8278(01)00181-7

[33] Cordoba J., Sanpedro F., Alonso J., and Rovira A.. 2002. ‘1H magnetic resonance in the study of hepatic encephalopathy in humans’, Metab Brain Dis, 17: 415–29. 12602517 10.1023/a:1021926405944

[34] Coupe P., Yger P., Prima S., Hellier P., Kervrann C., and Barillot C.. 2008. ‘An optimized blockwise nonlocal means denoising filter for 3-D magnetic resonance images’, IEEE Trans Med Imaging, 27: 425–41. 18390341 10.1109/TMI.2007.906087 PMC2881565

[35] Cox R. W. 1996. ‘AFNI: software for analysis and visualization of functional magnetic resonance neuroimages’, Comput Biomed Res, 29: 162–73. 8812068 10.1006/cbmr.1996.0014

[36] Dhiman R. K., Sawhney M. S., Chawla Y. K., Das G., Ram S., and Dilawari J. B.. 2000. ‘Efficacy of lactulose in cirrhotic patients with subclinical hepatic encephalopathy’, Dig Dis Sci, 45: 1549–52. 11007104 10.1023/a:1005556826152

[37] Diaz-Gomez D., Jover M., del-Campo J. A., Galindo A., and Romero-Gomez M.. 2011. ‘Experimental models for hepatic encephalopathy’, Rev Esp Enferm Dig, 103: 536–41. 22054269 10.4321/s1130-01082011001000006

[38] Donovan Jeremiah P., Schafer Daniel F., Shaw Byers W., and Sorrell Michael F.. 1998. ‘Cerebral oedema and increased intracranial pressure in chronic liver disease’, The Lancet, 351: 719–21. 10.1016/S0140-6736(97)07373-X 9504517

[39] Dreher W., and Leibfritz D.. 1999. ‘Detection of homonuclear decoupled in vivo proton NMR spectra using constant time chemical shift encoding: CT-PRESS’, Magn Reson Imaging, 17: 141–50. 9888407 10.1016/s0730-725x(98)00156-8

[40] Elder A., Gelein R., Silva V., Feikert T., Opanashuk L., Carter J., Potter R., Maynard A., Ito Y., Finkelstein J., and Oberdörster G.. 2006. ‘Translocation of inhaled ultrafine manganese oxide particles to the central nervous system’, Environ Health Perspect, 114: 1172–8. 16882521 10.1289/ehp.9030 PMC1552007

[41] Endicott J., Spitzer R. L., Fleiss J. L., and Cohen J.. 1976. ‘The global assessment scale. A procedure for measuring overall severity of psychiatric disturbance’, Arch Gen Psychiatry, 33: 766–71. 938196 10.1001/archpsyc.1976.01770060086012

[42] Fama R., Eisen J. C., Rosenbloom M. J., Sassoon S. A., Kemper C. A., Deresinski S., Pfefferbaum A., and Sullivan E. V.. 2007. ‘Upper and lower limb motor impairments in alcoholism, HIV infection, and their comorbidity’, Alcohol Clin Exp Res, 31: 1038–44. 17403062 10.1111/j.1530-0277.2007.00385.x

[43] Finkelstein Y., Zhang N., Fitsanakis V. A., Avison M. J., Gore J. C., and Aschner M.. 2008. ‘Differential deposition of manganese in the rat brain following subchronic exposure to manganese: a T1-weighted magnetic resonance imaging study’, Isr Med Assoc J, 10: 793–8. 19070289 PMC8988916

[44] First M.B., Spitzer R.L., Gibbon M., and Williams J.B.W.. 1998. “Structured Clinical Interview for DSMIV Axis I Disorders (SCID) Version 2.0.” In. New York, NY.: Biometrics Research Department, New York State Psychiatric Institute.

[45] Fischer-Smith T., Croul S., Adeniyi A., Rybicka K., Morgello S., Khalili K., and Rappaport J.. 2004. ‘Macrophage/microglial accumulation and proliferating cell nuclear antigen expression in the central nervous system in human immunodeficiency virus encephalopathy’, Am J Pathol, 164: 2089–99. 15161643 10.1016/S0002-9440(10)63767-4 PMC1615769

[46] Fitsanakis V. A., Zhang N., Anderson J. G., Erikson K. M., Avison M. J., Gore J. C., and Aschner M.. 2008. ‘Measuring brain manganese and iron accumulation in rats following 14 weeks of low-dose manganese treatment using atomic absorption spectroscopy and magnetic resonance imaging’, Toxicol Sci, 103: 116–24. 18234737 10.1093/toxsci/kfn019 PMC7910808

[47] Forton D. M., Allsop J. M., Main J., Foster G. R., Thomas H. C., and Taylor-Robinson S. D.. 2001. ‘Evidence for a cerebral effect of the hepatitis C virus’, Lancet, 358: 38–9. 11454379 10.1016/S0140-6736(00)05270-3

[48] Forton D. M., Hamilton G., Allsop J. M., Grover V. P., Wesnes K., O’Sullivan C., Thomas H. C., and Taylor-Robinson S. D.. 2008. ‘Cerebral immune activation in chronic hepatitis C infection: a magnetic resonance spectroscopy study’, J Hepatol, 49: 316–22. 18538439 10.1016/j.jhep.2008.03.022

[49] Forton D. M., Patel N., Prince M., Oatridge A., Hamilton G., Goldblatt J., Allsop J. M., Hajnal J. V., Thomas H. C., Bassendine M., Jones D. E., and Taylor-Robinson S. D.. 2004. ‘Fatigue and primary biliary cirrhosis: association of globus pallidus magnetisation transfer ratio measurements with fatigue severity and blood manganese levels’, Gut, 53: 587–92. 15016756 10.1136/gut.2003.016766 PMC1774014

[50] Forton D. M., Thomas H. C., Murphy C. A., Allsop J. M., Foster G. R., Main J., Wesnes K. A., and Taylor-Robinson S. D.. 2002. ‘Hepatitis C and cognitive impairment in a cohort of patients with mild liver disease’, Hepatology, 35: 433–9. 11826420 10.1053/jhep.2002.30688

[51] Fouad S. A., Esmat S., Omran D., Rashid L., and Kobaisi M. H.. 2012. ‘Noninvasive assessment of hepatic fibrosis in Egyptian patients with chronic hepatitis C virus infection’, World J Gastroenterol, 18: 2988–94. 22736923 10.3748/wjg.v18.i23.2988 PMC3380327

[52] Fregly A.R., Graybiel A., and Smith M.S.. 1972. ‘Walk on floor eyes closed (WOFEC): A new addition to an ataxia test battery’, Aerospace Medicine, 43: 395–99. 5045439

[53] Geissler A., Lock G., Fründ R., Held P., Hollerbach S., Andus T., Schölmerich J., Feuerbach S., and Holstege A.. 1997. ‘Cerebral abnormalities in patients with cirrhosis detected by proton magnetic resonance spectroscopy and magnetic resonance imaging’, Hepatology, 25: 48–54. 8985263 10.1053/jhep.1997.v25.pm0008985263

[54] Ginat D. T., and Meyers S. P.. 2012. ‘Intracranial lesions with high signal intensity on T1-weighted MR images: differential diagnosis’, Radiographics, 32: 499–516. 22411945 10.1148/rg.322105761

[55] Grover V. P., Pavese N., Koh S. B., Wylezinska M., Saxby B. K., Gerhard A., Forton D. M., Brooks D. J., Thomas H. C., and Taylor-Robinson S. D.. 2012. ‘Cerebral microglial activation in patients with hepatitis C: in vivo evidence of neuroinflammation’, J Viral Hepat, 19: e89–96. 22239531 10.1111/j.1365-2893.2011.01510.x

[56] Gupta R. K., Saraswat V. A., Poptani H., Dhiman R. K., Kohli A., Gujral R. B., and Naik S. R.. 1993. ‘Magnetic resonance imaging and localized in vivo proton spectroscopy in patients with fulminant hepatic failure’, Am J Gastroenterol, 88: 670–4. 8480729

[57] Han W., Zhang H., Han Y., and Duan Z.. 2020. ‘Cognition-tracking-based strategies for diagnosis and treatment of minimal hepatic encephalopathy’, Metab Brain Dis, 35: 869–81. 32495311 10.1007/s11011-020-00539-w PMC7354280

[58] Hanid M. A., Davies M., Mellon P. J., Silk D. B., Strunin L., McCabe J. J., and Williams R.. 1980. ‘Clinical monitoring of intracranial pressure in fulminant hepatic failure’, Gut, 21: 866–9. 6777264 10.1136/gut.21.10.866 PMC1419370

[59] Häussinger D., Laubenberger J., vom Dahl S., Ernst T., Bayer S., Langer M., Gerok W., and Hennig J.. 1994. ‘Proton magnetic resonance spectroscopy studies on human brain myo-inositol in hypoosmolarity and hepatic encephalopathy’, Gastroenterology, 107: 1475–80. 7926510 10.1016/0016-5085(94)90552-5

[60] Hegde Amogh N, Suyash Mohan, Narayan Lath, and Tchoyoson Lim CC. 2011. ‘Differential diagnosis for bilateral abnormalities of the basal ganglia and thalamus’, Radiographics, 31: 5–30. 21257930 10.1148/rg.311105041

[61] Henkelman R Mark, Watts John F, and Kucharczyk Walter. 1991. ‘High signal intensity in MR images of calcified brain tissue’, Radiology, 179: 199–206. 1848714 10.1148/radiology.179.1.1848714

[62] Hollingshead A. 1975. “Four-factor index of social status.” In. New Haven, CT: Department of Sociology, Yale University.

[63] Iglesias J. E., Liu C. Y., Thompson P. M., and Tu Z.. 2011. ‘Robust brain extraction across datasets and comparison with publicly available methods’, IEEE Trans Med Imaging, 30: 1617–34. 21880566 10.1109/TMI.2011.2138152

[64] Inoue E., Hori S., Narumi Y., Fujita M., Kuriyama K., Kadota T., and Kuroda C.. 1991. ‘Portal-systemic encephalopathy: presence of basal ganglia lesions with high signal intensity on MR images’, Radiology, 179: 551–5. 2014310 10.1148/radiology.179.2.2014310

[65] Jain L., Sharma B. C., Srivastava S., Puri S. K., Sharma P., and Sarin S.. 2013. ‘Serum endotoxin, inflammatory mediators, and magnetic resonance spectroscopy before and after treatment in patients with minimal hepatic encephalopathy’, J Gastroenterol Hepatol, 28: 1187–93. 23425082 10.1111/jgh.12160

[66] Kale R. A., Gupta R. K., Saraswat V. A., Hasan K. M., Trivedi R., Mishra A. M., Ranjan P., Pandey C. M., and Narayana P. A.. 2006. ‘Demonstration of interstitial cerebral edema with diffusion tensor MR imaging in type C hepatic encephalopathy’, Hepatology, 43: 698–706. 16557540 10.1002/hep.21114

[67] Khan M. A., and Anjum F.. 2021. ‘Portal-Systemic Encephalopathy.’ in, StatPearls (StatPearls Publishing LLC.: Treasure Island (FL)). 32965892

[68] Kim E. A., Cheong H. K., Choi D. S., Sakong J., Ryoo J. W., Park I., and Kang D. M.. 2007. ‘Effect of occupational manganese exposure on the central nervous system of welders: 1H magnetic resonance spectroscopy and MRI findings’, Neurotoxicology, 28: 276–83. 16824604 10.1016/j.neuro.2006.05.013

[69] Kim Y., Park J. K., Choi Y., Yoo C. I., Lee C. R., Lee H., Lee J. H., Kim S. R., Jeong T. H., Yoon C. S., and Park J. H.. 2005. ‘Blood manganese concentration is elevated in iron deficiency anemia patients, whereas globus pallidus signal intensity is minimally affected’, Neurotoxicology, 26: 107–11. 15527878 10.1016/j.neuro.2004.06.004

[70] Klatzo I. 1967. ‘Presidental address. Neuropathological aspects of brain edema’, J Neuropathol Exp Neurol, 26: 1–14. 5336776 10.1097/00005072-196701000-00001

[71] Klos K. J., Ahlskog J. E., Kumar N., Cambern S., Butz J., Burritt M., Fealey R. D., Cowl C. T., Parisi J. E., and Josephs K. A.. 2006. ‘Brain metal concentrations in chronic liver failure patients with pallidal T1 MRI hyperintensity’, Neurology, 67: 1984–9. 17159105 10.1212/01.wnl.0000247037.37807.76

[72] Kreis R., Ross B. D., Farrow N. A., and Ackerman Z.. 1992. ‘Metabolic disorders of the brain in chronic hepatic encephalopathy detected with H-1 MR spectroscopy’, Radiology, 182: 19–27. 1345760 10.1148/radiology.182.1.1345760

[73] Krieger D., Krieger S., Jansen O., Gass P., Theilmann L., and Lichtnecker H.. 1995. ‘Manganese and chronic hepatic encephalopathy’, Lancet, 346: 270–4. 7630246 10.1016/s0140-6736(95)92164-8

[74] Krieger S., Jauss M., Jansen O., Theilmann L., Geissler M., and Krieger D.. 1996. ‘Neuropsychiatric profile and hyperintense globus pallidus on T1-weighted magnetic resonance images in liver cirrhosis’, Gastroenterology, 111: 147–55. 8698193 10.1053/gast.1996.v111.pm8698193

[75] Kulisevsky J., Pujol J., Balanzó J., Junqué C., Deus J., Capdevilla A., and Villanueva C.. 1992. ‘Pallidal hyperintensity on magnetic resonance imaging in cirrhotic patients: clinical correlations’, Hepatology, 16: 1382–8. 1446893 10.1002/hep.1840160613

[76] Kumar R., Gupta R. K., Elderkin-Thompson V., Huda A., Sayre J., Kirsch C., Guze B., Han S., and Thomas M. A.. 2008. ‘Voxel-based diffusion tensor magnetic resonance imaging evaluation of low-grade hepatic encephalopathy’, J Magn Reson Imaging, 27: 1061–8. 18425846 10.1002/jmri.21342

[77] Kundra A., Jain A., Banga A., Bajaj G., and Kar P.. 2005. ‘Evaluation of plasma ammonia levels in patients with acute liver failure and chronic liver disease and its correlation with the severity of hepatic encephalopathy and clinical features of raised intracranial tension’, Clin Biochem, 38: 696–9. 15963970 10.1016/j.clinbiochem.2005.04.013

[78] Kwong Allison J., and Zahr Natalie M.. 2023. ‘Serum biomarkers of liver fibrosis identify globus pallidus vulnerability’, Neuroimage: Clinical, 37: 103333. 36868044 10.1016/j.nicl.2023.103333 PMC9996367

[79] Laubenberger J., Häussinger D., Bayer S., Gufler H., Hennig J., and Langer M.. 1997. ‘Proton magnetic resonance spectroscopy of the brain in symptomatic and asymptomatic patients with liver cirrhosis’, Gastroenterology, 112: 1610–6. 9136840 10.1016/s0016-5085(97)70043-x

[80] Lavender Charles A., Perisetti Abhilash, Salah Husam M., Sharma Tanya, Askew Emily A., Dranoff Jonathan A., and Thandassery Ragesh B.. 2020. ‘S1046 Non-Invasive Fibrosis Tests (APRI, FIB-4 and NFS) to Identify Advanced Fibrosis and Facilitate Referral to Liver Clinics’, Official journal of the American College of Gastroenterology | ACG, 115.

[81] Lee J. H., Seo D. W., Lee Y. S., Kim S. T., Mun C. W., Lim T. H., Min Y. I., and Suh D. J.. 1999. ‘Proton magnetic resonance spectroscopy (1H-MRS) findings for the brain in patients with liver cirrhosis reflect the hepatic functional reserve’, Am J Gastroenterol, 94: 2206–13. 10445551 10.1111/j.1572-0241.1999.01228.x

[82] Liang D., Bhatta S., Gerzanich V., and Simard J. M.. 2007. ‘Cytotoxic edema: mechanisms of pathological cell swelling’, Neurosurg Focus, 22: E2. 10.3171/foc.2007.22.5.3 PMC2740913 17613233

[83] Lidofsky S. D., Bass N. M., Prager M. C., Washington D. E., Read A. E., Wright T. L., Ascher N. L., Roberts J. P., Scharschmidt B. F., and Lake J. R.. 1992. ‘Intracranial pressure monitoring and liver transplantation for fulminant hepatic failure’, Hepatology, 16: 1–7. 1618463 10.1002/hep.1840160102

[84] Lin Z. H., Xin Y. N., Dong Q. J., Wang Q., Jiang X. J., Zhan S. H., Sun Y., and Xuan S. Y.. 2011. ‘Performance of the aspartate aminotransferase-to-platelet ratio index for the staging of hepatitis Crelated fibrosis: an updated meta-analysis’, Hepatology, 53: 726–36. 21319189 10.1002/hep.24105

[85] Lodi R., Tonon C., Stracciari A., Weiger M., Camaggi V., Iotti S., Donati G., Guarino M., Bolondi L., and Barbiroli B.. 2004. ‘Diffusion MRI shows increased water apparent diffusion coefficient in the brains of cirrhotics’, Neurology, 62: 762–6. 15007127 10.1212/01.wnl.0000113796.30989.74

[86] Long Zaiyang, Jiang Yue-Ming, Li Xiang-Rong, Fadel William, Xu Jun, Yeh Chien-Lin, Long Li-Ling, Luo Hai-Lan, Harezlak Jaroslaw, Murdoch James B., Zheng Wei, and Dydak Ulrike. 2014. ‘Vulnerability of welders to manganese exposure – A neuroimaging study’, Neurotoxicology, 45: 285–92. 24680838 10.1016/j.neuro.2014.03.007 PMC4177505

[87] Losowsky M. S., and Scott B. B.. 1973. ‘Hepatic encephalopathy’, Br Med J, 3: 279–81. 4579294 10.1136/bmj.3.5874.279 PMC1586730

[88] Ma R. E., Ward E. J., Yeh C. L., Snyder S., Long Z., Gokalp Yavuz F., Zauber S. E., and Dydak U.. 2018. ‘Thalamic GABA levels and occupational manganese neurotoxicity: Association with exposure levels and brain MRI’, Neurotoxicology, 64: 30–42. 28873337 10.1016/j.neuro.2017.08.013 PMC5891096

[89] Mader I., Rauer S., Gall P., and Klose U.. 2008. ‘(1)H MR spectroscopy of inflammation, infection and ischemia of the brain’, Eur J Radiol, 67: 250–7. 18407447 10.1016/j.ejrad.2008.02.033

[90] Maeda H., Sato M., Yoshikawa A., Kimura M., Sonomura T., Terada M., and Kishi K.. 1997. ‘Brain MR imaging in patients with hepatic cirrhosis: relationship between high intensity signal in basal ganglia on T1-weighted images and elemental concentrations in brain’, Neuroradiology, 39: 546–50. 9272489 10.1007/s002340050464

[91] Maffeo E., Montuschi A., Stura G., and Giordana M. T.. 2014. ‘Chronic acquired hepatocerebral degeneration, pallidal T1 MRI hyperintensity and manganese in a series of cirrhotic patients’, Neurol Sci, 35: 523–30. 23712371 10.1007/s10072-013-1458-x

[92] Mardini H., Smith F. E., Record C. O., and Blamire A. M.. 2011. ‘Magnetic resonance quantification of water and metabolites in the brain of cirrhotics following induced hyperammonaemia’, J Hepatol, 54: 1154–60. 21145802 10.1016/j.jhep.2010.09.030

[93] Martin R. C., Loring D. W., Meador K. J., and Lee G. P.. 1990. ‘The effects of lateralized temporal lobe dysfunction on formal and semantic word fluency’, Neuropsychologia, 28: 823–9. 2247208 10.1016/0028-3932(90)90006-a

[94] Mayer D., and Spielman D. M.. 2005. ‘Detection of glutamate in the human brain at 3 T using optimized constant time point resolved spectroscopy’, Magn Reson Med, 54: 439–42. 16032664 10.1002/mrm.20571

[95] Mayer D., Zahr N. M., Sullivan E. V., and Pfefferbaum A.. 2007. ‘In vivo metabolite differences between the basal ganglia and cerebellum of the rat brain detected with proton MRS at 3T’, Psychiatry Research-Neuroimaging, 154: 267–73. 10.1016/j.pscychresns.2006.11.005 PMC1892789 17346948

[96] McAndrews M. P., Farcnik K., Carlen P., Damyanovich A., Mrkonjic M., Jones S., and Heathcote E. J.. 2005. ‘Prevalence and significance of neurocognitive dysfunction in hepatitis C in the absence of correlated risk factors’, Hepatology, 41: 801–8. 15793853 10.1002/hep.20635

[97] McPherson S., Stewart S. F., Henderson E., Burt A. D., and Day C. P.. 2010. ‘Simple non-invasive fibrosis scoring systems can reliably exclude advanced fibrosis in patients with non-alcoholic fatty liver disease’, Gut, 59: 1265–9. 20801772 10.1136/gut.2010.216077

[98] Meng L. P., Chen Y. C., Li Y. H., Zhu J. S., and Ye J. L.. 2015. ‘Viability assessment of magnetic resonance spectroscopy for the detection of minimal hepatic encephalopathy severity’, Eur J Radiol, 84: 2019–23. 26170124 10.1016/j.ejrad.2015.06.027

[99] Miese F., Kircheis G., Wittsack H. J., Wenserski F., Hemker J., Modder U., Haussinger D., and Cohnen M.. 2006. ‘1H-MR spectroscopy, magnetization transfer, and diffusion-weighted imaging in alcoholic and nonalcoholic patients with cirrhosis with hepatic encephalopathy’, AJNR Am J Neuroradiol, 27: 1019–26. 16687536 PMC7975732

[100] Moon A. M., Singal A. G., and Tapper E. B.. 2020. ‘Contemporary Epidemiology of Chronic Liver Disease and Cirrhosis’, Clin Gastroenterol Hepatol, 18: 2650–66. 31401364 10.1016/j.cgh.2019.07.060 PMC7007353

[101] Morgan M. Y. 1998. ‘Cerebral magnetic resonance imaging in patients with chronic liver disease’, Metab Brain Dis, 13: 273–90. 10206820 10.1023/a:1020680624084

[102] Naegele T., Grodd W., Viebahn R., Seeger U., Klose U., Seitz D., Kaiser S., Mader I., Mayer J., Lauchart W., Gregor M., and Voigt K.. 2000. ‘MR imaging and (1)H spectroscopy of brain metabolites in hepatic encephalopathy: time-course of renormalization after liver transplantation’, Radiology, 216: 683–91. 10966695 10.1148/radiology.216.3.r00se27683

[103] Newland M. C., Ceckler T. L., Kordower J. H., and Weiss B.. 1989. ‘Visualizing manganese in the primate basal ganglia with magnetic resonance imaging’, Exp Neurol, 106: 251–8. 2591523 10.1016/0014-4886(89)90157-x

[104] Oriolo G., Egmond E., Mariño Z., Cavero M., Navines R., Zamarrenho L., Solà R., Pujol J., Bargallo N., Forns X., and Martin-Santos R.. 2018. ‘Systematic review with meta-analysis: neuroimaging in hepatitis C chronic infection’, Aliment Pharmacol Ther, 47: 1238–52. 29536563 10.1111/apt.14594

[105] Patel N., Forton D. M., Coutts G. A., Thomas H. C., and Taylor-Robinson S. D.. 2000. ‘Intracellular pH measurements of the whole head and the basal ganglia in chronic liver disease: a phosphorus-31 MR spectroscopy study’, Metab Brain Dis, 15: 223–40. 11206591 10.1007/BF02674531

[106] Patel N., White S., Dhanjal N. S., Oatridge A., and Taylor-Robinson S. D.. 2004. ‘Changes in brain size in hepatic encephalopathy: a coregistered MRI study’, Metab Brain Dis, 19: 431–45. 15554433 10.1023/b:mebr.0000043987.09022.e3

[107] Pautler Robia G., Mongeau Raymond, and Jacobs Russell E.. 2003. ‘In vivo trans-synaptic tract tracing from the murine striatum and amygdala utilizing manganese enhanced MRI (MEMRI)’, Magnetic Resonance in Medicine, 50: 33–39. 12815676 10.1002/mrm.10498

[108] Pfefferbaum A., Zahr N. M., Sassoon S. A., Kwon D., Pohl K. M., and Sullivan E. V.. 2018. ‘Accelerated and Premature Aging Characterizing Regional Cortical Volume Loss in Human Immunodeficiency Virus Infection: Contributions From Alcohol, Substance Use, and Hepatitis C Coinfection’, Biol Psychiatry Cogn Neurosci Neuroimaging, 3: 844–59. 30093343 10.1016/j.bpsc.2018.06.006 PMC6508083

[109] Pokorska-Śpiewak Maria, Aleksandra Stańska-Perka Jolanta Popielska, Agnieszka Ołdakowska Urszula Coupland, Zawadka Konrad, Szczepańska-Putz Małgorzata, and Marczyńska Magdalena. 2017. ‘Prevalence and predictors of liver disease in HIV-infected children and adolescents’, Sci Rep, 7: 12309. 28951598 10.1038/s41598-017-11489-2 PMC5615053

[110] Poordad F. F. 2007. ‘Review article: the burden of hepatic encephalopathy’, Aliment Pharmacol Ther, 25 Suppl 1: 3–9. 10.1111/j.1746-6342.2006.03215.x 17295846

[111] Poveda M. J., Bernabeu A., Concepcion L., Roa E., de Madaria E., Zapater P., Perez-Mateo M., and Jover R.. 2010. ‘Brain edema dynamics in patients with overt hepatic encephalopathy A magnetic resonance imaging study’, Neuroimage, 52: 481–7. 20451628 10.1016/j.neuroimage.2010.04.260

[112] Prakash R., and Mullen K. D.. 2010. ‘Mechanisms, diagnosis and management of hepatic encephalopathy’, Nat Rev Gastroenterol Hepatol, 7: 515–25. 20703237 10.1038/nrgastro.2010.116

[113] Prasad S., Dhiman R. K., Duseja A., Chawla Y. K., Sharma A., and Agarwal R.. 2007. ‘Lactulose improves cognitive functions and health-related quality of life in patients with cirrhosis who have minimal hepatic encephalopathy’, Hepatology, 45: 549–59. 17326150 10.1002/hep.21533

[114] Pujol A., Pujol J., Graus F., Rimola A., Peri J., Mercader J. M., García-Pagan J. C., Bosch J., Rodés J., and Tolosa E.. 1993. ‘Hyperintense globus pallidus on T1-weighted MRI in cirrhotic patients is associated with severity of liver failure’, Neurology, 43: 65–9. 8423913 10.1212/wnl.43.1_part_1.65

[115] Pujol J., Kulisevsky J., Moreno A., Deus J., Alonso J., Balanzo J., Marti-Vilalta J. L., and Capdevila A.. 1996. ‘Neurospectroscopic alterations and globus pallidus hyperintensity as related magnetic resonance markers of reversible hepatic encephalopathy’, Neurology, 47: 1526–30. 8960739 10.1212/wnl.47.6.1526

[116] Rama Rao K. V., and Norenberg M. D.. 2014. ‘Glutamine in the pathogenesis of hepatic encephalopathy: the trojan horse hypothesis revisited’, Neurochem Res, 39: 593–8. 23277414 10.1007/s11064-012-0955-2 PMC4737090

[117] Reichardt J. L., Dirks M., Wirries A. K., Pflugrad H., Nösel P., Haag K., Lanfermann H., Wedemeyer H., Potthoff A., Weissenborn K., and Ding X. Q.. 2022. ‘Brain metabolic and microstructural alterations associated with hepatitis C virus infection, autoimmune hepatitis and primary biliary cholangitis’, Liver Int, 42: 842–52. 34719118 10.1111/liv.15093

[118] Ridola L., Faccioli J., Nardelli S., Gioia S., and Riggio O.. 2020. ‘Hepatic Encephalopathy: Diagnosis and Management’, J Transl Int Med, 8: 210–19. 33511048 10.2478/jtim-2020-0034 PMC7805282

[119] Rohlfing T., Brandt R., Menzel R., and Maurer C. R. Jr. 2004. ‘Evaluation of atlas selection strategies for atlas-based image segmentation with application to confocal microscopy images of bee brains’, Neuroimage, 21: 1428–42. 15050568 10.1016/j.neuroimage.2003.11.010

[120] Rose C., Butterworth R. F., Zayed J., Normandin L., Todd K., Michalak A., Spahr L., Huet P. M., and Pomier-Layrargues G.. 1999. ‘Manganese deposition in basal ganglia structures results from both portal-systemic shunting and liver dysfunction’, Gastroenterology, 117: 640–4. 10464140 10.1016/s0016-5085(99)70457-9

[121] Ross B. D., Jacobson S., Villamil F., Korula J., Kreis R., Ernst T., Shonk T., and Moats R. A.. 1994. ‘Subclinical hepatic encephalopathy: proton MR spectroscopic abnormalities’, Radiology, 193: 457–63. 7972763 10.1148/radiology.193.2.7972763

[122] Ryan Jennifer M., and Shawcross Debbie L.. 2015. ‘Hepatic encephalopathy’, Medicine (Baltimore), 43: 679–82.

[123] Ryu A. J., Rahimi R. S., and Leise M. D.. 2020. ‘The Current Hepatic Encephalopathy Pipeline’, J Clin Exp Hepatol, 10: 377–85. 32655239 10.1016/j.jceh.2020.01.001 PMC7335727

[124] Sadananthan S. A., Zheng W., Chee M. W., and Zagorodnov V.. 2010. ‘Skull stripping using graph cuts’, Neuroimage, 49: 225–39. 19732839 10.1016/j.neuroimage.2009.08.050

[125] Sherman K. E., Peters M. G., and Thomas D.. 2017. ‘Human immunodeficiency virus and liver disease: A comprehensive update’, Hepatol Commun, 1: 987–1001. 30838978 10.1002/hep4.1112 PMC5721407

[126] Shin H. W., and Park H. K.. 2017. ‘Recent Updates on Acquired Hepatocerebral Degeneration’, Tremor Other Hyperkinet Mov (N Y), 7: 463. 28975044 10.7916/D8TB1K44 PMC5623760

[127] Shoreibah M., Anand B. S., and Singal A. K.. 2014. ‘Alcoholic hepatitis and concomitant hepatitis C virus infection’, World J Gastroenterol, 20: 11929–34. 25232227 10.3748/wjg.v20.i34.11929 PMC4161778

[128] Singal A. K., and Anand B. S.. 2007. ‘Mechanisms of synergy between alcohol and hepatitis C virus’, J Clin Gastroenterol, 41: 761–72. 17700425 10.1097/MCG.0b013e3180381584

[129] Singhal A., Nagarajan R., Hinkin C. H., Kumar R., Sayre J., Elderkin-Thompson V., Huda A., Gupta R. K., Han S. H., and Thomas M. A.. 2010. ‘Two-dimensional MR spectroscopy of minimal hepatic encephalopathy and neuropsychological correlates in vivo’, J Magn Reson Imaging, 32: 35–43. 20578008 10.1002/jmri.22216

[130] Skinner H. A., and Sheu W. J.. 1982. ‘Reliability of alcohol use indices. The Lifetime Drinking History and the MAST’, J Stud Alcohol, 43: 1157–70. 7182675 10.15288/jsa.1982.43.1157

[131] Smith S. M. 2002. ‘Fast robust automated brain extraction’, Hum Brain Mapp, 17: 143–55. 12391568 10.1002/hbm.10062 PMC6871816

[132] Spahr L., Butterworth R. F., Fontaine S., Bui L., Therrien G., Milette P. C., Lebrun L. H., Zayed J., Leblanc A., and Pomier-Layrargues G.. 1996. ‘Increased blood manganese in cirrhotic patients: relationship to pallidal magnetic resonance signal hyperintensity and neurological symptoms’, Hepatology, 24: 1116–20. 8903385 10.1002/hep.510240523

[133] Spreen Otfried. 1977. ‘Neurosensory center comprehensive examination for aphasia’, Neuropsychological Laboratory.

[134] Stein Eva, Monica Cruz-Lemini Jose Altamirano, Ndugga Nambi, Couper David, Abraldes Juan G, and Bataller Ramon. 2016. ‘Heavy daily alcohol intake at the population level predicts the weight of alcohol in cirrhosis burden worldwide’, J Hepatol, 65: 998–1005. 27392424 10.1016/j.jhep.2016.06.018

[135] Sterling R. K., Lissen E., Clumeck N., Sola R., Correa M. C., Montaner J., Sulkowski M S., Torriani F. J., Dieterich D. T., Thomas D. L., Messinger D., Nelson M., and Apricot Clinical Investigators. 2006. ‘Development of a simple noninvasive index to predict significant fibrosis in patients with HIV/HCV coinfection’, Hepatology, 43: 1317–25. 16729309 10.1002/hep.21178

[136] Sullivan E. V., Deshmukh A., Desmond J. E., Lim K. O., and Pfefferbaum A.. 2000. ‘Cerebellar volume decline in normal aging, alcoholism, and Korsakoff’s syndrome: relation to ataxia’, Neuropsychology, 14: 341–52. 10928737 10.1037//0894-4105.14.3.341

[137] Sullivan E. V., Zahr N. M., Sassoon S. A., Thompson W. K., Kwon D., Pohl K. M., and Pfefferbaum A.. 2018. ‘The Role of Aging, Drug Dependence, and Hepatitis C Comorbidity in Alcoholism Cortical Compromise’, JAMA Psychiatry. 10.1001/jamapsychiatry.2018.0021 PMC5875381 29541774

[138] Takahashi H., Koehler R. C., Brusilow S. W., and Traystman R. J.. 1991. ‘Inhibition of brain glutamine accumulation prevents cerebral edema in hyperammonemic rats’, Am J Physiol, 261: H825–9. 1679605 10.1152/ajpheart.1991.261.3.H825

[139] Takeda A., Sawashita J., and Okada S.. 1998. ‘Manganese concentration in rat brain: manganese transport from the peripheral tissues’, Neurosci Lett, 242: 45–8. 9510001 10.1016/s0304-3940(98)00006-8

[140] Taylor-Robinson S. D., Oatridge A., Hajnal J. V., Burroughs A. K., McIntyre N., and deSouza N. M.. 1995. ‘MR imaging of the basal ganglia in chronic liver disease: correlation of T1-weighted and magnetisation transfer contrast measurements with liver dysfunction and neuropsychiatric status’, Metab Brain Dis, 10: 175–88. 7675015 10.1007/BF01991864

[141] Taylor-Robinson S. D., Sargentoni J., Oatridge A., Bryant D. J., Hajnal J. V., Marcus C. D., Seery J. P., Hodgson H. J., and deSouza N. M.. 1996. ‘MR imaging and spectroscopy of the basal ganglia in chronic liver disease: correlation of T1-weighted contrast measurements with abnormalities in proton and phosphorus-31 MR spectra’, Metab Brain Dis, 11: 249–68. 8869945 10.1007/BF02237962

[142] Thames April D, Castellon Steven A, Singer Elyse J, Nagarajan Rajakumar, Sarma Manoj K, Smith Jason, Thaler Nicholas S, Truong Jonathan Hien, Schonfeld Daniel, and Thomas M Albert. 2015. ‘Neuroimaging abnormalities, neurocognitive function, and fatigue in patients with hepatitis C’, Neurology-Neuroimmunology Neuroinflammation, 2. 10.1212/NXI.0000000000000059 PMC4299885 25610883

[143] Thandassery R. B., Al Kaabi S., Soofi M. E., Mohiuddin S. A., John A. K., Al Mohannadi M., Al Ejji K., Yakoob R., Derbala M. F., Wani H., Sharma M., Al Dweik N., Butt M. T., Kamel Y. M., Sultan K., Pasic F., and Singh R.. 2016. ‘Mean Platelet Volume, Red Cell Distribution Width to Platelet Count Ratio, Globulin Platelet Index, and 16 Other Indirect Noninvasive Fibrosis Scores: How Much Do Routine Blood Tests Tell About Liver Fibrosis in Chronic Hepatitis C?’, J Clin Gastroenterol, 50: 518–23. 26974762 10.1097/MCG.0000000000000489

[144] Thomas M. A., Huda A., Guze B., Curran J., Bugbee M., Fairbanks L., Ke Y., Oshiro T., Martin P., and Fawzy F.. 1998. ‘Cerebral 1H MR spectroscopy and neuropsychologic status of patients with hepatic encephalopathy’, AJR Am J Roentgenol, 171: 1123–30. 9763008 10.2214/ajr.171.4.9763008

[145] Trites R.L. 1977. ‘The Grooved Pegboard Test.’ in, Neuropsychological Test Manual (Royal Ottawa Hospital: Ontario, Canada).

[146] Valcour V. G., Rubin L. H., Obasi M. U., Maki P. M., Peters M. G., Levin S., Crystal H. A., Young M. A., Mack W. J., Cohen M. H., Pierce C. B., Adimora A. A., and Tien P. C.. 2016. ‘Liver Fibrosis Linked to Cognitive Performance in HIV and Hepatitis C’, J Acquir Immune Defic Syndr, 72: 266–73. 26885801 10.1097/QAI.0000000000000957 PMC4911304

[147] Cauter Van, Sofie Mariasavina Severino, Ammendola Rosamaria, Brecht Van Berkel Hrvoje Vavro, van den Hauwe Luc, and Rumboldt Zoran. 2020. ‘Bilateral lesions of the basal ganglia and thalami (central grey matter)—pictorial review’, Neuroradiology, 62: 1565–605. 32761278 10.1007/s00234-020-02511-y PMC7405775

[148] Vaquero J., Chung C., Cahill M. E., and Blei A. T.. 2003. ‘Pathogenesis of hepatic encephalopathy in acute liver failure’, Semin Liver Dis, 23: 259–69. 14523679 10.1055/s-2003-42644

[149] Ware Athol J., D’Agostino Anthony N., and Combes Burton. 1971. ‘Cerebral Edema: A Major Complication of Massive Hepatic Necrosis’, Gastroenterology, 61: 877–84. 5125688

[150] Ware J. E. Jr., Bayliss M. S., Mannocchia M., and Davis G. L.. 1999. ‘Health-related quality of life in chronic hepatitis C: impact of disease and treatment response. The Interventional Therapy Group’, Hepatology, 30: 550–5. 10421667 10.1002/hep.510300203

[151] Watanabe A., Shiota T., and Tsuji T.. 1992. ‘Cerebral edema during hepatic encephalopathy in fulminant hepatic failure’, J Med, 23: 29–38. 1573340

[152] Weber R., Sabin C. A., Friis-Moller N., Reiss P., El-Sadr W. M., Kirk O., Dabis F., Law M. G., Pradier C., De Wit S., Akerlund B., Calvo G., Monforte Ad, Rickenbach M., Ledergerber B., Phillips A. N., and Lundgren J. D.. 2006. ‘Liver-related deaths in persons infected with the human immunodeficiency virus: the D:A:D study’, Arch Intern Med, 166: 1632–41. 16908797 10.1001/archinte.166.15.1632

[153] Wedler F. C., and Denman R. B.. 1984. ‘Glutamine synthetase: the major Mn(II) enzyme in mammalian brain’, Curr Top Cell Regul, 24: 153–69. 6149889 10.1016/b978-0-12-152824-9.50021-6

[154] Wedler F. C., Denman R. B., and Roby W. G.. 1982. ‘Glutamine synthetase from ovine brain is a manganese(II) enzyme’, Biochemistry, 21: 6389–96. 6129892 10.1021/bi00268a011

[155] Weissenborn K., Bokemeyer M., Ahl B., Fischer-Wasels D., Giewekemeyer K., van den Hoff J., Köstler H., and Berding G.. 2004. ‘Functional imaging of the brain in patients with liver cirrhosis’, Metab Brain Dis, 19: 269–80. 15554422 10.1023/b:mebr.0000043976.17500.8e

[156] Weissenborn K., Ehrenheim C., Hori A., Kubicka S., and Manns M. P.. 1995. ‘Pallidal lesions in patients with liver cirrhosis: clinical and MRI evaluation’, Metab Brain Dis, 10: 219–31. 8830282 10.1007/BF02081027

[157] Wijdicks E. F. 2016. ‘Hepatic Encephalopathy’, N Engl J Med, 375: 1660–70. 27783916 10.1056/NEJMra1600561

[158] Wijdicks E. F., Plevak D. J., Rakela J., and Wiesner R. H.. 1995. ‘Clinical and radiologic features of cerebral edema in fulminant hepatic failure’, Mayo Clin Proc, 70: 119–24. 7845036 10.4065/70.2.119

[159] Williams J. B., and Rabkin J. G.. 1991. ‘The concurrent validity of items in the Quality-of-Life Index in a cohort of HIV-positive and HIV-negative gay men’, Control Clin Trials, 12: 129s–41s. 1663850 10.1016/s0197-2456(05)80018-2

[160] Yamamoto H., Konno H., Yamamoto T., Ito K., Mizugaki M., and Iwasaki Y.. 1987. ‘Glutamine synthetase of the human brain: purification and characterization’, J Neurochem, 49: 603–9. 2885398 10.1111/j.1471-4159.1987.tb02906.x

[161] Young S.W. 1984. Nuclear magnetic resonance imaging: Basic principles (Raven Press; Reprint edition (January 1, 1984)).

[162] Zahr N. M., Mayer D., Rohlfing T., Chanraud S., Gu M., Sullivan E. V., and Pfefferbaum A.. 2013. ‘In vivo glutamate measured with magnetic resonance spectroscopy: behavioral correlates in aging’, Neurobiol Aging, 34: 1265–76. 23116877 10.1016/j.neurobiolaging.2012.09.014 PMC3545108

[163] Zahr N. M., Mayer D., Rohlfing T., Sullivan E. V., and Pfefferbaum A.. 2014. ‘Imaging neuroinflammation? A perspective from MR spectroscopy’, Brain Pathol, 24: 654–64. 25345895 10.1111/bpa.12197 PMC4493672

[164] Zahr N. M., Pohl K. M., Kwong A. J., Sullivan E. V., and Pfefferbaum A.. 2021. ‘Preliminary Evidence for a Relationship between Elevated Plasma TNFα and Smaller Subcortical White Matter Volume in HCV Infection Irrespective of HIV or AUD Comorbidity’, Int J Mol Sci, 22. 10.3390/ijms22094953 PMC8124321 34067023

[165] Zaitout Z., Romanowski C., Karunasaagarar K., Connolly D., and Batty R.. 2014. ‘A review of pathologies associated with high T1W signal intensity in the basal ganglia on Magnetic Resonance Imaging’, Pol J Radiol, 79: 126–30. 24900164 10.12659/PJR.890043 PMC4043538

[166] Zeneroli Maria L, Giorgio Cioni, Girolamo Crisi, Cinzia Vezzelli, and Ezio Ventura. 1991. ‘Globus pallidus alterations and brain atrophy in liver cirrhosis patients with encephalopathy: an MR imaging study’, Magn Reson Imaging, 9: 295–302. 1881246 10.1016/0730-725x(91)90414-h

[167] Zhou Y., Eid T., Hassel B., and Danbolt N. C.. 2020. ‘Novel aspects of glutamine synthetase in ammonia homeostasis’, Neurochem Int, 140: 104809. 32758585 10.1016/j.neuint.2020.104809

